# Locoregional Breast Cancer Recurrences After Ablatio Mammae and Primary Reconstruction

**DOI:** 10.3390/jcm15010326

**Published:** 2026-01-01

**Authors:** Constance Valette, Alexandra Anker, Michael Gerken, Stephan Seitz, Monika Klinkhammer-Schalke, Silvan Eisenmann, Marc Ruewe, Philipp Unbehaun, Lukas Prantl, Vanessa Brébant

**Affiliations:** 1 Department of Plastic, Hand and Reconstructive Surgery, University Hospital Regensburg, Franz-Josef-Strauß-Allee 11, D-93053 Regensburg, Germany; valette.constancemariecaroline@gmail.com (C.V.); silvan.eisenmann@ukr.de (S.E.); marc.ruewe@ukr.de (M.R.); philipp.unbehaun@ukr.de (P.U.); lukas.prantl@ukr.de (L.P.); 2 Tumor Center—Center for Quality Assurance and Health Services Research, University of Regensburg, Am Biopark 9, D-93053 Regensburg, Germany; michael.gerken@klinik.uni-regensburg.de (M.G.); monika.klinkhammer-schalke@ur.de (M.K.-S.); 3 Department of Obstetrics and Gynecology, Caritas Saint Josef Hospital, University of Regensburg, Landshuter Strasse 65, D-93055 Regensburg, Germany; sseitz@csj.de

**Keywords:** breast cancer, locoregional recurrence, cumulative recurrence rate, overall survival, recurrence-free survival, autologous reconstruction, allogeneic reconstruction, primary reconstruction, dormant cancer cells

## Abstract

**Background/Objectives:** Breast cancer is the most common cancer among women worldwide. Surgical treatments include breast-conserving therapy (BCT) and mastectomy, often followed by reconstruction, but the impact of reconstruction on locoregional recurrence (LRR) remains unclear. This study evaluated LRR, survival, and risk factors following primary breast reconstruction performed simultaneously with mastectomy compared with mastectomy without reconstruction. **Methods:** This population-based, retrospective cohort included 2475 women with breast cancer treated between 2004 and 2018 at the Tumor Center and Caritas St. Josef Hospital in Regensburg, Germany. Patients were grouped into not primarily reconstructed, primary autologous reconstruction, primary allogeneic reconstruction, and primary combined reconstruction. Overall survival (OS), recurrence-free survival (RFS), and cumulative recurrence rates (CRR) were assessed using Kaplan–Meier methods and Cox proportional hazards models adjusted for age, nodal status, tumor biology, and adjuvant therapies. **Results:** Of 14,046 eligible cases, 2475 met inclusion criteria: no primary reconstruction (87%), autologous reconstruction (3.1%), allogeneic reconstruction (9.0%), and combined reconstruction (0.4%). Patients undergoing reconstruction were younger and more likely to receive chemotherapy. The 5-year OS was 71.8% without primary reconstruction, 82.1% after autologous reconstruction, and 90.0% after allogeneic reconstruction. Allogeneic reconstruction was associated with improved OS (HR 0.570, *p* = 0.015) and RFS (HR 0.669, *p* = 0.039), whereas autologous reconstruction was associated with higher hazards of LRR and distant metastases compared to no primary reconstruction. **Conclusions:** The 5-year cumulative LRR was 5.2%, 13.5%, and 4.8%, respectively. Immediate allogeneic reconstruction after mastectomy was therefore associated with favorable survival and recurrence outcomes, while autologous reconstruction was linked to higher LRR and distant metastasis rates in this cohort. The retrospective design, small autologous subgroup, and absence of detailed lifestyle and metabolic data are important limitations of these findings. These associations likely reflect differences in tumor stage, biology, and unmeasured risk factors, and should be interpreted as hypothesis generating. Prospective multicenter studies with detailed risk profiling are needed to clarify the oncologic safety of different reconstructive strategies.

## 1. Introduction

Breast cancer remains the most prevalent cancer among women worldwide, with a steadily increasing incidence [1]. In Germany alone, approximately 70.550 new cases are diagnosed annually, mirroring global trends [2]. Surgical treatment is a cornerstone of breast cancer management, often complemented by systemic therapies such as chemotherapy, radiation, hormonal therapy, and targeted treatments [3,4,5,6,7,8,9,10,11]. Surgical approaches include BCT and mastectomy [12,13,14,15].

Breast reconstruction following mastectomy is an integral component of treatment for many patients. The main goal of surgical breast cancer therapy is to cure the disease and prevent recurrence, while primary reconstruction not only restores breast aesthetics but has also been proven to significantly enhance quality of life [16]. Reconstruction options are broadly categorized into autologous and allogeneic methods. Autologous reconstruction uses tissue from donor sites such as the abdomen, back, or thighs, with the deep inferior epigastric perforator (DIEP) flap nowadays often considered the gold standard [17,18,19,20,21]. Allogeneic reconstruction is based on silicone implants, sometimes combined with acellular dermal matrices or other meshes to optimize outcomes. Both approaches have demonstrated benefits for health-related quality of life (HRQoL), including improved satisfaction with breast appearance, psychosocial well-being, body image, and self-esteem [22].

Despite these benefits, breast reconstruction also carries potential risks and complications. Locoregional recurrences (LRRs) are a critical concern in breast cancer treatment, encompassing the reappearance of cancer in the breast, chest wall, or regional lymph nodes. The risk of LRRs is influenced by tumor biology, including receptor status, surgical margin status, lymphovascular invasion, nodal positivity, patient age and family history, tumor necrosis, tumor markers, and the timing of systemic therapy [3,4,5,8,9,23,24,25,26,27,28,29,30,31,32,33]. Emerging evidence suggests that surgical trauma and the associated wound healing processes might also contribute to tumor recurrence through mechanisms such as neoangiogenesis and the release of growth factors [34,35,36].

The purpose of this study is to compare the risks associated with primary autologous and allogeneic reconstruction following mastectomy and to analyze potential risk factors for locoregional recurrence. By examining the incidence and survival outcomes of LRRs in patients undergoing primary autologous or allogeneic breast reconstruction versus those without primary reconstruction, this study aims to provide a more nuanced understanding of the role of reconstructive surgery in breast cancer management.

## 2. Materials and Methods

### 2.1. Study Design and Patient Cohort

This study employed a retrospective, population-based cohort design utilizing data from the Tumor Center and the Caritas St. Josef Hospital in Regensburg, Bavaria, Germany. The Tumor Center, a regional cancer registry of high quality, collects comprehensive cancer data from Upper Palatinate and Lower Bavaria, including contributions from the University Hospital Regensburg, 53 regional hospitals, and over 1500 practicing physicians. It also integrates mortality data from regional registries and health offices. Medical records provided detailed information on diagnoses, therapies, disease progression, long-term follow-ups, locoregional and distant recurrences, and mortality.

### 2.2. Patient Inclusion and Exclusion Criteria

The dataset comprised 2475 breast cancer patients treated with mastectomy between January 2004 and December 2018, including those who underwent primary breast reconstruction and those who did not. Comparative analyses were conducted for the following groups:

- Mastectomy without primary reconstruction
- Mastectomy with primary autologous reconstruction
- Mastectomy with primary allogeneic reconstruction
- Mastectomy with combined primary autologous and allogeneic reconstruction

These four categories were chosen because they reflect common clinical pathways and allow separate assessment of purely autologous and purely implant-based procedures, while keeping combined procedures in a distinct group, as these patients often have more complex reconstructions and risk profiles. Delayed and secondary reconstructions were excluded to focus on immediate reconstruction performed at the time of mastectomy.

Other exclusion criteria encompassed male patients, tumors other than adenocarcinomas, stage IV and M1 tumors, reconstructions following BCT, and cases with residual tumor status (R1/2 or RX/not available).

### 2.3. Statistical Analysis

The study evaluated five-year OS, RFS, LRR, metastatic recurrence rate, and CRR from diagnosis to the first recorded event. Kaplan–Meier survival curves were used to estimate OS and RFS, with censoring applied at the last follow-up, recurrence date, or time of death.

To assess the impact of surgical approaches and additional covariates on survival, univariable and multivariable Cox proportional hazard models were employed. Multivariable regression analyses, adjusted for confounding factors (e.g., age, nodal status, histological grading, lymphovascular invasion, hormone receptor status, HER2 status, Ki-67, and adjuvant therapies), compared OS and RFS across different reconstruction methods. Statistical significance was defined as a two-sided *p*-value < 0.05 and confidence intervals (CI) excluding 1.0. All analyses were conducted using IBM SPSS Statistics 25 (SPSS Inc., Chicago, IL, USA).

In addition to the variables available in the tumor registry, several potentially relevant clinical risk factors were not systematically documented and therefore could not be included in the multivariable models. These variables include body mass index and other markers of metabolic syndrome, smoking and alcohol consumption, and perioperative factors such as blood transfusions. As high body mass index and lifestyle-related factors are associated with both eligibility for specific reconstructive procedures and oncologic outcomes, their absence may have introduced residual confounding and contributed to the observed differences between reconstruction groups.

## 3. Results

### 3.1. Inclusion and Exclusion Characteristics

The study initially included 14,046 primary breast cancer patients diagnosed between 2004 and 2018. After applying exclusion criteria, 2475 patients remained for analysis. These patients underwent surgery and were classified as UICC Stage I–III, X/na, and M0. Among the final cohort, 87% (n = 2156) had no primary reconstruction, 3.1% (n = 76) underwent autologous reconstruction, and 9% (n = 222) had allogeneic reconstruction.

### 3.2. Patient and Tumor Characteristics

Reconstruction was more frequently performed in younger, premenopausal patients, who were also more likely to receive chemotherapy compared to patients who were not primarily reconstructed. The mean age for patients without primary reconstruction was 67.0 years, compared to 49.8 years in the autologous group and 50.3 years in the allogeneic group. Tumor-specific characteristics also varied between groups; patients in the autologous reconstruction cohort had a higher incidence of T4 breast cancer (7.9%) compared to those undergoing allogeneic reconstruction (0.9%). HER2/-neu (human epidermal growth factor receptor 2) negativity was reported at 72.7% for not primarily reconstructed, 56.6% for autologous reconstruction and 68.5% for allogeneic reconstruction. Locoregional characteristics, such as lymph vessel invasion, were less frequent in the autologous (23.7%) and allogeneic (25.2%) groups compared to patients without primary reconstruction (41.5%).

### 3.3. Cumulative Recurrence, 5-Year Overall Survival and Recurrence-Free Survival

**Figure 1:**
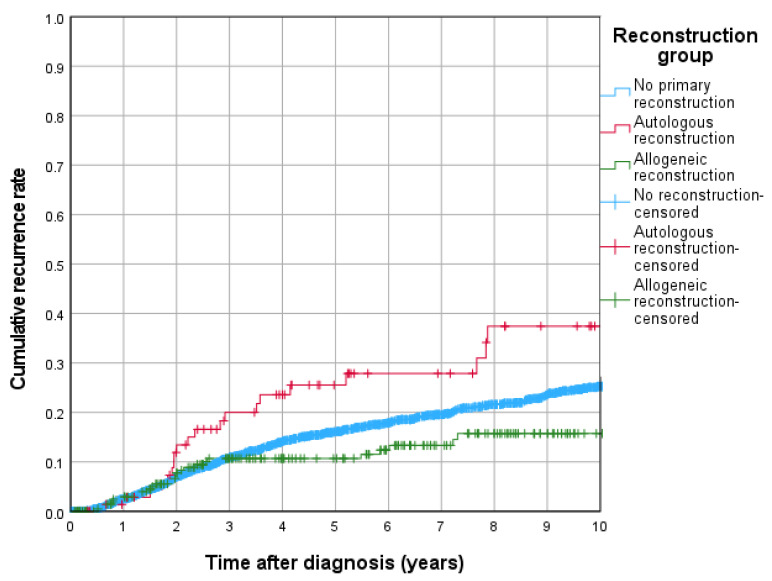
Cumulative recurrence rates of reconstruction groups.

The 5-year OS rate was highest in allogeneic reconstruction (90.0%), followed by autologous reconstruction (82.1%) and no primary reconstruction (71.8%). The 5-year CRR was highest in autologous reconstruction at 25.5%, significantly exceeding both allogeneic reconstruction at 10.7% (*p* = 0.005) and no primary reconstruction at 16.1%, with allogeneic reconstruction demonstrating the most favorable outcome in terms of recurrence risk (Figure 1).

The underlying numbers of patients and recurrent events for each reconstruction group are summarized in Table 1; in particular, the autologous reconstruction group comprises 76 patients with 20 recurrent events, which helps explain the pronounced step-like appearance of its Kaplan–Meier curve.

**Figure 2:**
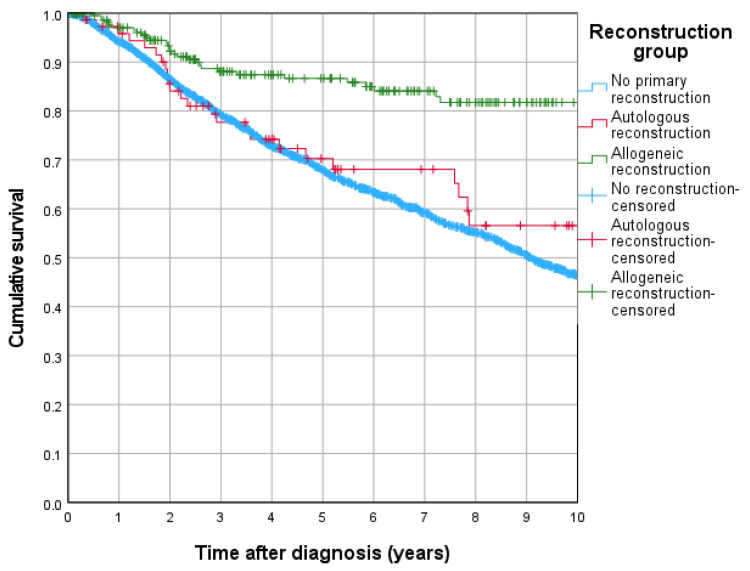
Recurrence free survival rates of reconstruction groups.

Following this, the 5-year RFS rates were 86.7% for allogeneic reconstruction, 70.3% for autologous reconstruction, and 67.9% for no primary reconstruction. Kaplan–Meier Figure 2 survival curves illustrate the statistical significance of allogeneic reconstruction over both autologous reconstruction (*p* < 0.001) and no primary reconstruction (*p* < 0.001).

### 3.4. Cumulative Locoregional Recurrence and Locoregional Recurrence Free Survival

**Figure 3:**
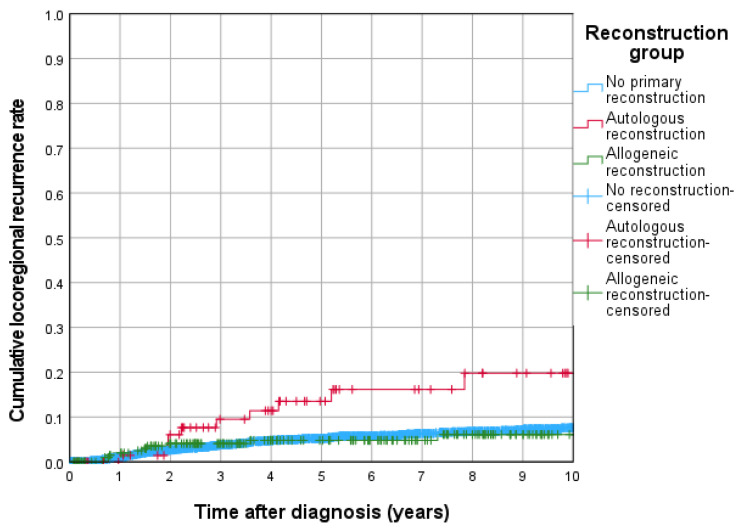
Cumulative locoregional recurrence rates of reconstruction groups.

The cumulative 5-year locoregional recurrence rates were highest in the autologous reconstruction group (13.5%), followed by the no primary reconstruction group (5.2%) and the allogeneic reconstruction group (4.8%). Kaplan–Meier analysis revealed a significantly higher risk of locoregional recurrence in the autologous group compared to both the allogeneic group (*p* = 0.024) and no primary reconstruction group (*p* = 0.002) (Figure 3).

**Figure 4:**
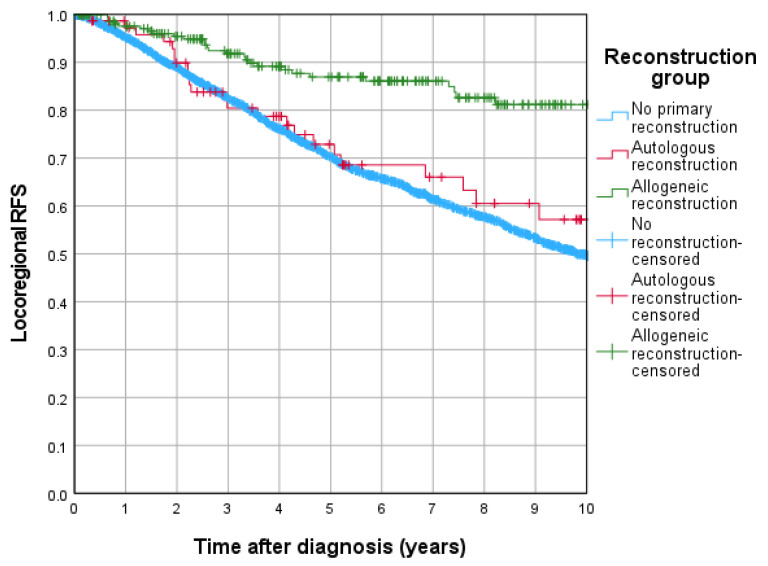
Locoregional recurrence free survival rates for reconstruction groups.

The 5-year RFS rates were 70.2% for patients without primary reconstruction, 72.9% for those undergoing autologous reconstruction, and no events were recorded for the allogeneic reconstruction group (Figure 4). Notably, allogeneic reconstruction demonstrated a significantly better outcome with a *p*-value of <0.001 compared to both autologous reconstruction and no primary reconstruction.

### 3.5. Multivariable Cox Regression Analysis

To adjust for potential confounders, multivariable Cox regression analyses were performed for each of the long-term outcomes (Table 2). The results showed that allogeneic reconstruction significantly reduced the hazard of death compared to no primary reconstruction (HR = 0.570, 95% CI: 0.363–0.896; *p* = 0.015). In contrast, autologous reconstruction did not significantly affect OS compared to no primary reconstruction (HR = 1.437, 95% CI: 0.895–2.306; *p* = 0.133).

RFS analysis revealed that allogeneic reconstruction was associated with a significantly lower hazard for death and recurrence compared to no primary reconstruction (HR = 0.669, 95% CI: 0.457–0.980; *p* = 0.039). Conversely, autologous reconstruction was associated with a higher hazard for death and recurrence compared to no primary reconstruction (HR = 1.690, 95% CI: 1.124–2.541; *p* = 0.012).

Regarding CRR, autologous reconstruction was associated with a significantly higher hazard compared to no primary reconstruction (HR = 2.156, 95% CI: 1.333–3.486; *p* = 0.002), while allogeneic reconstruction showed no significant difference (HR = 0.889, 95% CI: 0.585–1.353; *p* = 0.583).

With respect to cumulative locoregional recurrence rate, autologous reconstruction was related with a significantly higher hazard compared to no primary reconstruction (HR = 3.016, 95% CI: 1.472–6.178; *p* = 0.003), while allogeneic reconstruction again showed no significant difference (HR = 1.143, 95% CI: 0.577–2.265; *p* = 0.701).

Additionally, autologous reconstruction was linked to an increased hazard for distant metastases compared to no primary reconstruction (HR = 2.070, 95% CI: 1.187–3.610; *p* = 0.010), whereas allogeneic reconstruction did not significantly increase the hazard for distant metastases (HR = 0.900, 95% CI: 0.559–1.449; *p* = 0.663).

## 4. Discussion

The findings of this study highlight several noteworthy results, particularly in relation to breast reconstruction and its influence on outcomes of breast cancer patients following mastectomy. From the findings, it appears that breast reconstructions tend to be performed more on younger patients, while postmenopausal women more frequently decline the procedure. This tendency may reflect differing priorities, risk perceptions, and considerations regarding quality of life among these patient groups.

One of the key findings is that allogeneic immediate reconstruction demonstrated better outcomes compared to both not primarily reconstructed and autologous reconstruction, even after adjusting for the available risk factors. Specifically, allogeneic reconstruction was associated with improved OS and RFS. This result warrants critical discussion. The placement of a silicone implant after mastectomy appears to reduce the risk of recurrence and positively influence survival. It is plausible that important risk-inducing factors are missing from the tumor registry data. Examples of such factors include BMI, metabolic syndrome, genetic predispositions, smoking status or alcohol consumption [34,37,38,39,40,41,42,43,44,45,46,47]. Obesity increases cancer risk through reprogrammed adipocytes that release pro-tumorigenic factors, promoting growth and metastasis [34]. Nicotine enhances ALDH-positive breast cancer cell populations and CSC proliferation, potentially driving tumor progression [39]. Alcohol modestly increases breast cancer risk, linked to breast density and genetic factors, but its impact on recurrence and survival is inconclusive [38]. These factors could significantly influence patient outcomes and introduce residual confounding, which may explain some of the observed differences between reconstruction types.

Another crucial point is the limited number of cases with autologous reconstruction (n = 76), which must be acknowledged as a limitation when interpreting oncological safety. This relatively small sample size increases the risk of selection bias, as such procedures are often offered to patients with more favorable prognostic features, such as lower comorbidity burden, better overall health, and higher likelihood of treatment adherence.

Regarding oncologic parameters, the TNM classification across the reconstruction groups revealed relevant differences. Patients with autologous reconstruction more frequently presented with T4 tumors (7.9%) compared to allogeneic reconstruction (0.9%), suggesting more locally advanced disease in this subgroup. However, N1 nodal status was relatively balanced across all groups (25.4% in no primary reconstruction, 26.3% in autologous, and 22.1% in allogeneic). The majority of patients across all groups were in UICC stage II, indicating comparability of tumor burden.

Interestingly, the percentage of HER2-negative tumors was highest in the not primarily reconstructed group (72.7%), whereas autologous and allogeneic reconstruction groups had lower rates of HER2 negativity (56.6% and 68.5%, respectively). HER2-positive tumors, while historically considered more aggressive, now benefit from targeted therapy options, potentially altering their prognostic weight [48]. Ki-67, a marker of proliferative activity, was under 25% in nearly half of all cases, with slightly lower values in the not primarily reconstructed group. A Ki-67 over 25% was significantly associated with worse overall survival in the multivariable model, highlighting its role as an independent risk factor [32].

The distribution of molecular subtypes adds another important layer: luminal B tumors were more frequent in the autologous reconstruction group (35.5%) compared to the not primarily reconstructed group (30.5%) and the allogeneic group (27.9%). These tumors are known to have a more aggressive course than luminal A tumors, potentially influencing the treatment plan and reconstruction decision [49].

Taken together, these tumor and subtype distributions suggest that the autologous reconstruction group comprised patients with a less favorable oncologic profile at baseline. The higher proportion of T4 tumors, the larger share of biologically more aggressive subtypes, and the likely enrichment of patients with higher body mass index, who are often considered better candidates for autologous flaps and less suitable for implant-based reconstruction, may all have contributed to the increased risk of recurrence and metastasis in this group. It therefore appears plausible that patient and tumor selection, together with unmeasured risk factors such as obesity and metabolic comorbidities, largely explain the higher LRR and distant recurrence rates observed after autologous reconstruction, rather than the reconstructive technique itself being inherently oncologically unsafe.

Therapeutically, chemotherapy was more commonly administered to patients undergoing reconstruction (53.9% autologous, 54.1% allogeneic) than those without primary reconstruction (39.6%). This may indicate a tendency to offer reconstruction to patients receiving more aggressive treatment. Interestingly, endocrine therapy was also frequently applied in all groups, with slightly higher rates in the autologous group (55.3%) compared to allogeneic (52.3%). Radiotherapy, although a critical component of breast cancer treatment, was less frequently administered in the reconstruction groups (23.7% autologous, allogeneic 23.9%) than in the not primarily reconstructed group (35.6%). This discrepancy might reflect treatment planning decisions favoring delayed reconstruction when radiotherapy is anticipated.

These therapy distributions highlight the need to interpret outcome differences in the context of multimodal treatment strategies. In particular, the impact of adjuvant and neoadjuvant therapies should be considered in future prospective analyses, especially since neoadjuvant therapy can alter staging and influence reconstruction eligibility and timing.

The heightened inflammatory response and trauma induced by extensive combined surgical procedures, including autologous breast reconstruction, may also contribute to the observed outcomes [35,36,50,51]. Increased inflammation and trauma could eliminate homeostatic constraints on metastatic site formation, potentially supporting the development of breast cancer metastases. Furthermore, angiocrine factors and specific extracellular matrix proteins produced by endothelial cells (ECs) may influence the dormancy and activation of disseminated breast cancer cells, with significant implications for cancer progression and therapeutic resistance. A recent study revealed that inflammatory processes drive lung-residing endothelial cells to produce secreted proteins that are crucial components of the vascular niche in metastasis [52]. The role of angiogenesis in surgery-driven escape from dormancy of breast cancer cells for pre-menopausal patients is particularly noteworthy. These factors collectively highlight the potential impact on cancer progression and resistance to therapeutic treatments [53].

While the present registry study cannot directly test these mechanisms, they provide a biologically plausible framework for understanding how complex reconstruction might interact with pre-existing micrometastatic disease.

However, it is also essential to consider the inflammatory processes associated with allogeneic reconstructions, particularly the potential development of Breast Implant-Associated Anaplastic Large Cell Lymphoma (BIA-ALCL). This malignancy arises in an inflammatory microenvironment characterized by lymphocyte infiltration and a Th1/Th17 phenotype, often involving JAK/STAT pathway mutations [54]. Evidence suggests that bacterial biofilms on implant surfaces may trigger chronic inflammation, driving tumorigenesis. While implant shells themselves do not directly stimulate tumor growth, they may act as passive carriers for bacteria, with biofilm-derived lipopolysaccharide (LPS) interacting with TLR4 to promote inflammatory cytokine release and tumor progression [55]. These findings underscore the complex interplay of inflammation and bacterial presence in the pathogenesis of BIA-ALCL.

Other factors, such as the potential immunosuppressive effect of homologous blood transfusions during surgical treatment, may also play a role. Studies on colorectal cancer patients have shown that transfusions are associated with increased locoregional recurrence rates, likely due to immunomodulation and reduced resistance to circulating tumor cells [56]. While direct parallels to breast cancer surgery require further investigation, this serves as a reminder of the complex interplay between surgical interventions and oncologic outcomes.

This registry-based study comes with several limitations. The retrospective design, incomplete documentation, and incomplete recording of key risk factors limit the ability to draw definitive conclusions. The low cumulative recurrence rate of local recurrences observed in this study may reflect the relatively short follow-up. Additionally, the absence of crucial confounder information, such as precise data on metabolic factors, genetic predispositions, and lifestyle factors like BMI, smoking status, alcohol consumption, and nicotine use, highlights the inherent limitations of relying on pre-existing databases. It is plausible that important risk-inducing factors are missing from the tumor registry data.

First, the retrospective, registry-based design is subject to residual confounding and potential selection biases in the choice of reconstructive procedure. Second, the autologous reconstruction subgroup was relatively small (n = 76), which limits statistical power, results in wide confidence intervals for some estimates, and precludes definitive conclusions regarding the oncologic safety of autologous techniques. Third, key risk factors such as body mass index, metabolic syndrome, smoking and alcohol use, and detailed genetic information were not available and could not be incorporated into the multivariable models. Finally, follow up duration and imaging schedules were not fully standardized across all contributing centers, which may have influenced the detection and timing of locoregional recurrences. These constraints necessitate cautious interpretation of the findings and underscore the need for prospective, well controlled multicenter studies.

Despite these challenges, register-based clinical and epidemiological data offer several advantages, including large sample sizes and population-based and unselected Real World Data. Adjusting for certain confounders available for the entire population can enhance the accuracy of estimated associations. However, the reliance on pre-collected data introduces risks of misclassification and residual confounding. Careful attention to these biases is essential to ensure the reliability of findings in register-based research.

It is evident that more comprehensive analyses are needed to confirm these findings and address the limitations of this study. Prospective cohort studies with detailed data collection on confounders and longer follow-up periods could provide more robust insights. Furthermore, exploring the molecular and biological mechanisms underlying the observed differences in outcomes between reconstruction types is critical for advancing our understanding and guiding clinical practice.

## 5. Conclusions

This study has revealed novel findings regarding primary breast reconstruction and its impact on outcomes following mastectomy in breast cancer patients. Allogeneic reconstruction showed better OS and RFS compared to no primary reconstruction and autologous reconstruction, even after risk adjustment. However, these results must be interpreted with caution due to the retrospective design, the inherent limitations of registry-based data, incomplete recording of confounders, and the relatively low incidence of local recurrences. To comprehensively address these questions and validate the observed associations, future research should prioritize prospective, multicenter study design.

The heightened inflammatory response and trauma associated with extensive surgical procedures, particularly autologous reconstruction, could contribute to the observed differences in locoregional and metastatic recurrence rates.

Future research should aim to better define the patient groups for whom primary reconstruction may be safe and beneficial. Delayed reconstruction after mastectomy, combined with rigorous follow-up care, may help to minimize potential risks of locoregional recurrences. Larger, prospective studies with comprehensive data collection are essential to validate these findings and provide clearer guidance for clinical decision making.

In this large, population-based cohort of women undergoing mastectomy, immediate allogeneic reconstruction was associated with favorable overall and recurrence free survival compared with no primary reconstruction, whereas patients treated with autologous reconstruction experienced higher rates of locoregional recurrence, distant metastases, and overall recurrence. These associations likely reflect differences in tumor stage, biology, and unmeasured risk factors, including body mass index and other comorbidities, that influence reconstructive decision making. The results should therefore be interpreted as hypothesis generating rather than as proof of a causal detrimental effect of autologous reconstruction. Future prospective multicenter studies with standardized staging, detailed risk profiling, and harmonized follow up protocols are needed to clarify the oncologic safety of different reconstructive strategies and to guide individualized decision making for patients considering immediate breast reconstruction after mastectomy.

## Figures and Tables

**Table 1:** Number of patients and recurrent events according to reconstruction groups.

Reconstruction Group	Total n	n of Events
No reconstruction	2156	373
Autologous reconstruction	76	20
Allogeneic reconstruction	222	26
Overall	2475	421

**Table 2:** Results from univariable and multivariable Cox regression analyses by reconstruction for different endpoints. Multivariable Cox models were adjusted for age at diagnosis, nodal status, histological grade, lymphovascular invasion, hormone receptor status, HER2 status, Ki-67, and adjuvant systemic and radiation therapies. Ref., reference group.

	Univariable Cox-Regression				Multivariable Cox-Regression			
	*p*	HR	Lower Bound 95%-CI	Upper Bound 95%-CI	*p*	HR	Lower Bound 95%-CI	Upper Bound 95%-CI
Overall survival								
No primary reconstruction	<0.001	Ref.			0.050	Ref.		
Autologous reconstruction	0.078	0.664	0.421	1.046	0.133	1.437	0.895	2.306
Allogeneic reconstruction	<0.001	0.258	0.168	0.398	0.015	0.570	0.363	0.896
Recurrence free survival								
No primary reconstruction	<0.001	Ref.			0.010	Ref.		
Autologous reconstruction	0.482	0.870	0.589	1.284	0.012	1.690	1.124	2.541
Allogeneic reconstruction	<0.001	0.335	0.233	0.482	0.039	0.669	0.457	0.980
Cumulative recurrence rate (total)								
No primary reconstruction	0.061	Ref.			0.016	Ref.		
Autologous reconstruction	0.032	1.637	1.044	2.568	0.002	2.156	1.333	3.486
Allogeneic reconstruction	0.099	0.716	0.481	1.065	0.583	0.889	0.585	1.353
Cumulative locoregional recurrence rate								
No primary reconstruction	0.054	Ref.			0.035	Ref.		
Autologous reconstruction	0.003	2.633	1.379	5.028	0.003	3.016	1.472	6.178
Allogeneic reconstruction	0.992	0.997	0.537	1.851	0.701	1.143	0.577	2.265
Cumulative metastatic recurrence rate								
No primary reconstruction	0.224	Ref.			0.101	Ref.		
Autologous reconstruction	0.076	1.602	0.952	2.693	0.010	2.070	1.187	3.610
Allogeneic reconstruction	0.196	0.741	0.471	1.167	0.663	0.900	0.559	1.449

## Data Availability

Inquiries regarding the raw data can be addressed to the corresponding author at any time.

## Abbreviations

The following abbreviations are used in this manuscript:

BCT	Breast conserving therapy
DIEP	Deep inferior epigastric perforator
LRR	Locoregional recurrence rate
OS	Overall survival
RFS	Recurrence-free survival
CRR	Cumulative recurrence rate
CI	Confidence interval
HR	Hazard ratio
EC	Endothelial cell
BIA-ALCL	Breast Implant-Associated Anaplastic Large Cell Lymphoma
